# Integrative analysis of transcriptomics and metabolomics reveals the protective effect and mechanism of salidroside on testicular ischemia-reperfusion injury

**DOI:** 10.3389/fphar.2024.1377836

**Published:** 2024-05-16

**Authors:** Ya Ping Jiang, Bao Gui Liu, Yi Dang, Lin Jie Liu, Yang Pang, Xiao Dong Bai, Feng Sun, Tian Hong Kang, Zheng Hang Zhao

**Affiliations:** ^1^ Department of Pharmacology, School of Basic Medical Sciences, Xi’an Jiaotong University Health Science Center, Shaanxi, China; ^2^ Department of Pharmacy, Xianyang Hospital of Yan’an University, Shaanxi, China; ^3^ Department of Clinical Research, Xianyang Hospital of Yan’an University, Shaanxi, China; ^4^ Department of Science and Education, Xianyang Hospital of Yan’an University, Shaanxi, China

**Keywords:** salidroside, protective effects, ischemia-reperfusion injury, ferroptosis, Nfr2/HO-1/GPX4 signaling pathway

## Abstract

Testicular torsion is a critical urologic condition for which testicular detorsion surgery is considered irreplaceable as well as the golden method of reversal. However, the surgical treatment is equivalent to a blood reperfusion process, and no specific drugs are available to treat blood reperfusion injuries. Salidroside (SAL) is one of the main effective substances in rhodiola, which has been shown to have antioxidant and antiapoptosis activities. This study was designed to determine whether SAL exerted a protective effect on testicular ischemia-reperfusion (I/R) injury. In this study, the I/R injury model of the testes and reoxygenation (OGD/R) model were used for verification, and SAL was administered at doses of 100 mg/kg and 0.05 mmol/L, respectively. After the experiments, the testicular tissue and TM4 Sertoli cells were collected for histopathologic and biochemical analyses. The results revealed that SAL improves the structure of testicular tissue and regulates the oxidation–antioxidation system. To further understand the molecular mechanisms of SAL in treating testicular I/R injuries, transcriptomics and metabonomics analyses were integrated. The results show that the Nfr2/HO-1/GPX4/ferroptosis signaling pathway is enriched significantly, indicating that it may be the main regulatory pathway for SAL in the treatment of testicular I/R injuries. Thereafter, transfection with Nrf2 plasmid–liposome was used to reverse verify that the Nfr2/HO-1/GPX4/ferroptosis signaling pathway was the main pathway for SAL anti-testicular I/R injury treatment. Thus, it is suggested that SAL can protect against testicular I/R injuries by regulating the Nfr2/HO-1/GPX4 signaling pathway to inhibit ferroptosis and that SAL may be a potential drug for the treatment of testicular I/R injuries.

## Highlights

• This pilot study examines the potential effects of salidroside on testicular ischemia-reperfusion injuries via transcriptomics and metabonomics.
• Salidroside is shown to have potential beneficial effects on testicular ischemia-reperfusion injuries.
• Salidroside intervention is shown to prevent ferroptosis by regulating the Nfr2/HO-1/GPX4 signaling pathway.
• Salidroside can be potentially used to treat testicular ischemia-reperfusion injuries.

## 1 Introduction

Testicular torsion is a critical urologic disease caused by winding of the spermatic cord around the testis (Guazzone and Lustig, 2023; Lacy et al., 2023). The incidence of testicular torsion is 1 in 4,000 in male individuals younger than 25 years of age, whereas the prevalence of testicular torsion out of all acute scrotal conditions is 25%–50% (Baskovic et al., 2022). Despite timely detorsion, testicular atrophy may still occur in 9.1%–73.3% of patients in long-term follow up, seriously affecting their spermatogenic functions (Wei and Huang, 2022). In addition, testicular atrophy, sperm quality decline, and ultimately decreased fertility were observed in an animal model of testicular torsion.

The damage caused by testicular torsion detorsion is called an ischemia-reperfusion (I/R) injury (Chen et al., 2023), and I/R injuries occur after incidents of acute ischemia. Upon blood flow restoration, the testes appear to be injured by I/R, which is the primary pathophysiology of testicular torsion detorsion (Wei and Huang, 2022; Almarzouq and Al, 2023; Alnajem and Al, 2023). In the I/R process, there is formation of excess reactive oxygen species (ROS), such as superoxide anions, nitric oxide, hydrogen peroxide, and hydroxyl radicals, in the affected tissue; these ROS are highly reactive and can damage the cellular constituents, such as lipids, proteins, DNA, and carbohydrates. Testicular tissue is very sensitive to ROS, eventually resulting in loss of testicular cell vitality and even cell death. When oxidative stress occurs in the testicular tissue, the nuclear factor erythroid-2 related factor-2 (Nrf2) dissociates from Keap1 and transfers into the nucleus, thereby regulating the expressions of downstream antioxidant proteases, such as heme oxygenase-1 (HO-1) and further exerting antioxidant stresses (Ma, 2013; Sies et al., 2017). Nrf2 is a key transcription factor for regulating ROS and is an important regulator for maintaining intracellular redox balance. Nrf2 can also induce and regulate the compositions and expressions of antioxidant proteins, reduce the production of ROS, and maintain the redox stability of the body (Wang T. et al, 2020; Chen, 2022; Lee et al., 2024). The key iron-storing proteins ferritin light chain/ferritin heavy chain (FTL/FTH1) and glutathione peroxide 4 (GPX4) are regulated by Nrf2. Therefore, targeting Nrf2 is a feasible disease-intervention strategy to regulate lipid peroxidation (LPO) and ferroptosis (Long et al., 2023; Qiu et al., 2023).

Ferroptosis is a new type of programmed cell death characterized by LPO and excessive oxidative stress. In recent years, GPX4 has been regarded as a star molecule for inhibiting ferroptosis and related diseases; its main mechanism of action is the role of an antioxidant with GSH as an auxiliary factor. Thus, GSH/GPX4 is the core pathway for regulating ferroptosis *in vivo* (Dong et al., 2023; Zhou et al., 2023; Xia et al., 2024). Many studies have shown that ferroptosis is very important in testicular I/R injuries and that inhibiting oxidative stress and ferroptosis can effectively improve such injuries (Li et al., 2018; Xiang et al., 2023; Zhao et al., 2024). Although many studies have been conducted on testicular I/R injuries, clinical treatment methods are still lacking. Once testicular torsion occurs, testicular detorsion surgery is still considered the golden method of reversing the condition, and the surgical approach is considered undeniable. However, the surgical treatment is equivalent to a blood reperfusion process, and no specific drugs are available to treat the injuries (Wei and Huang, 2022; Abdel et al., 2023; Chen et al., 2023). Therefore, the development of effective therapeutic drugs is a core problem in treating testicular I/R injuries.

Salidroside (SAL) is one of the main effective substances in rhodiola and has many important biological characteristics, including antioxidant and antiapoptosis activities (Liu et al., 2023; Wang et al., 2023; Yang et al., 2023). Previous studies have shown that SAL can improve the symptoms of dry eye by inhibiting oxidative stress injuries (Liang et al., 2023). SAL can also protect dihydrotestosterone-induced granular tumor cells by regulating the Nrf2/HO-1 pathway to inhibit oxidative stress (Ji et al., 2022). In addition, recent studies have shown the protective effects of SAL on spermatogenesis in streptozotocin induced type 1 diabetic male mice by inhibiting oxidative-stress-mediated blood–testis barrier damage (Jiang et al., 2020). All of these studies have shown that SAL has the strongest effects on antioxidant defenses that can support increase in mitochondrial permeability, scavenging of free radicals, and inhibition of lipid peroxide. Accordingly, it is hypothesized in this study that SAL may improve testicular I/R injuries by inhibiting oxidative stress. In this work, the protective effects of SAL on testicular I/R injuries are investigated systematically. By combining RNA-seq transcriptome sequencing and metabolomics analysis, the specific mechanism of action of SAL is also determined in the hope of identifying potential new drugs and their mechanisms for the treatment of testicular I/R injuries.

## 2 Materials and methods

### 2.1 Animals

A total of 18 healthy adult mice provided by the Hu’nan Slack Jingda Experimental Animal Co., Ltd., were used in this study. All mice weighed 20 ± 2 g, with the ambient temperature being 22–26°C and ambient humidity maintained at 45%–65%. The lights were controlled to achieve 12 h of day and 12 h of night conditions, and all experiments were performed under pathogen-free conditions with free access to food and water.

### 2.2 Establishment of the I/R model and drug treatment

**FIGURE 1:**
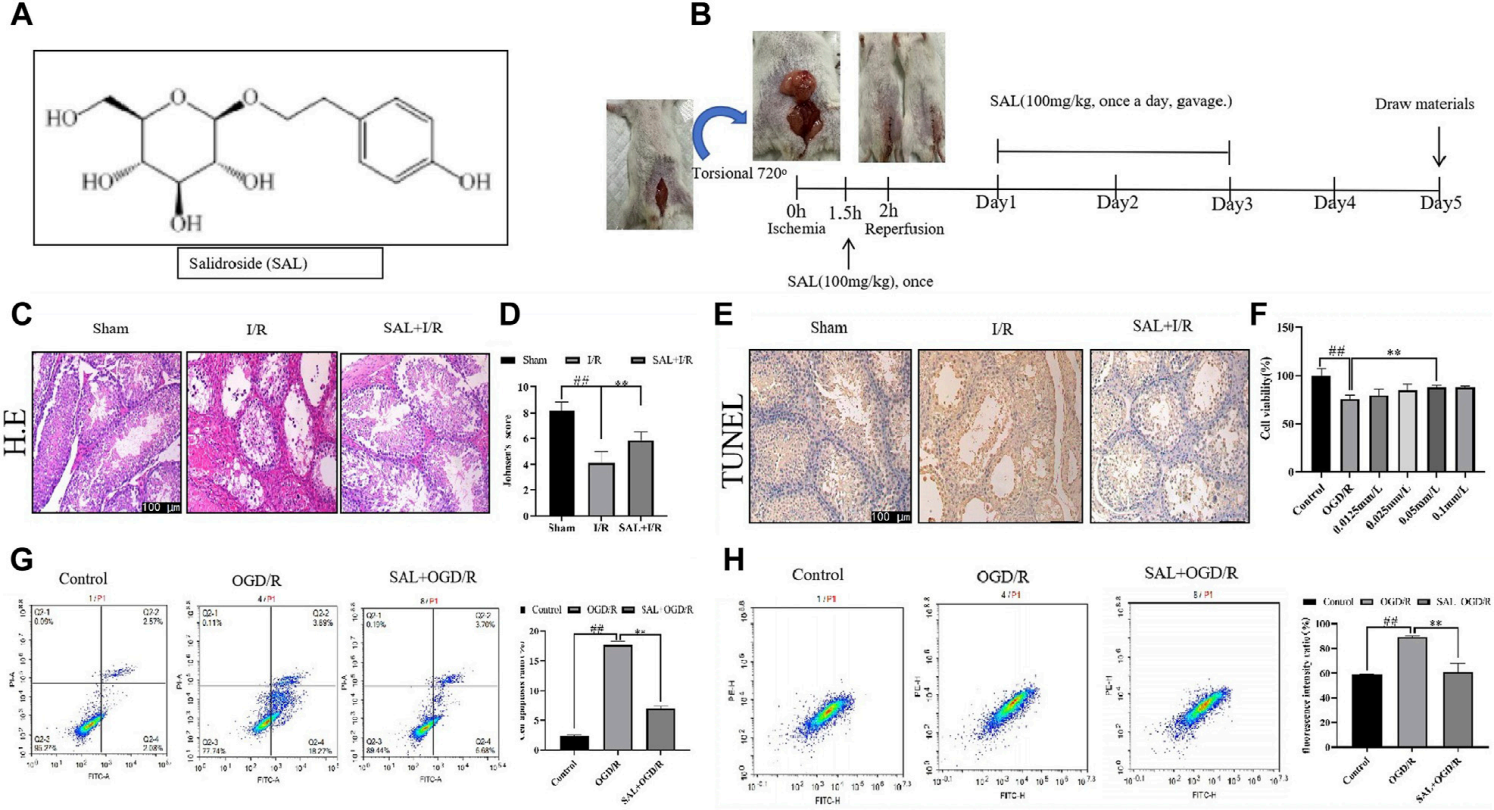
Protective effect of salidroside (SAL) on testicular I/R injury. **(A)** Chemical structure of SAL; **(B)** experimental design; **(C)** representative H&E staining photomicrographs of the seminiferous tubules of the testis at ×200 magnification (scale bar = 100 μm); **(D)** Johnsen’s testicular score; **(E)** Tunel staining; **(F)** effect of SAL on the viability of TM4 cells; **(G)** cell apoptosis ratio; **(H)** fluorescence intensity ratio. Each value indicates the mean ± standard deviation (*n = 6*)*.* ^*##*^ *p < 0.01* vs. sham group or control group; *p < 0.05; **p < 0.01 vs. I/R group or OGD/R group.

The animal model of I/R was established as per the Turner method. First, an intraperitoneal injection of 2% pentobarbital sodium (50 mg/kg) was administered for anesthesia, and a small incision was made in the lower abdomen to free the left testis; then, the testicular lead was cut off and surrounding fascia was separated up to the head of the epididymis. The left testis was then rotated 720° clockwise around the spermatic cord; the tunica albuginea was sutured and fixed to prevent automatic reduction, and the scrotum was sutured. After torsion for 2 h, the testis was reset and fixed. In the control group, a sham operation was performed, where the left scrotum was cut and testis was exposed freely without being twisted before the scrotum was closed by suture. Both groups were given the same volume of normal saline for treatment. In the I/R model group, the left testis was twisted by 720°, and it was reset and fixed 2 h later. In the I/R model + SAL group, 100 mg/kg of SAL (Beijing Zhongke Quality Inspection Biotechnology Co., Ltd., Beijing, China, CAS, 10338-51-9, purity 99.94%) was administered by gavage 30 min before torsion reduction, once a day on the first, second, and third days after operation, and samples were obtained on the fifth day after operation (Figure 1A, B).

### 2.3 Cell treatment

TM4 mouse Sertoli cells were purchased from the type culture collection of the Chinese Academy of Sciences and cultured in DMEM-F12 medium with 2.5% fetal bovine serum (FBS) and 5% horse serum (HS) at 37°C and 5% CO_2_ in a box. After reaching 80%–90% confluence, the TM4 cells were shifted to a serum-free DMEM-F12 medium for 24 h. Conventional culture of the TM4 cells was performed next by adding sugar-free 90% DMEM/F12 + 5% HS + 2.5% FBS + 1% P/S solution, placing the cells in a three-gas incubator (1% O_2_ + 94% N_2_ + 5% CO_2_) for culture to achieve oxygen–glucose deprivation, and changing to the conventional culture solution after 2 h to achieve reoxygenation (OGD/R) over the next 3 h. Then, 2 mL of PBS and 0.3 g of SAL were dissolved in a storage solution of concentration 0.5 mol/L, followed by filtration with a 0.22-μm filter membrane and sterilization. Before the experiments, the cell culture medium was diluted to prepare drug-containing cultures of concentrations 10, 1, 0.5, 0.1, 0.05, 0.25, and 0.0125 mmol/L. To verify the mechanism of action, the cells were divided into three groups as control, OGD/R, and SAL (0.05 mmol/L) groups. After 24 h, the cells were harvested for subsequent experiments, namely, immunofluorescence, protein blot, and quantitative polymerases chain reaction (qPCR).

### 2.4 CCK8 determination

The prepared 96-well plate was placed in 5% CO_2_ at 37°C in an oven overnight to achieve full adherence to the walls; then, the culture solution was removed from the 96-well plate, SAL was added in a gradient at 100 μL per well, and the plate was placed in an incubator for 48 h. Next, the culture plate was retrieved and approximately 10 μL of CCK8 was added to each well before incubating in an incubator for 2 h; the sample absorbance (OD) was then measured at 450 nm using a microplate reader, and the cell survival rate was calculated according to the formula: cell survival rate (%) = [(OD of the administration group – OD of the blank well)/(OD of the normal group – OD of the blank well)] × 100%.

### 2.5 Biochemical analysis of oxidative stress

The ROS level was assayed using a fluorescence spectrophotometer and 2,7-dichlorofluorescein diacetate (DCFH-DA) according to the assay kit (Nanjing Jiancheng Bioengineering Institute, Nanjing, China). In brief, appropriate amounts of the testicular cells were pre-incubated with DCFH-DA (10 μM) at 37°C to allow the DCFH-DA to be incorporated into any membrane-bound vesicles. The conversion of DCFH to DCF (green fluorescence) was evaluated using a fluorescence spectrophotometer at an excitation wavelength of 485 nm and emission at 525 nm. Data were expressed in terms of the fluorescence intensity values. The level of malondialdehyde (MDA) and the enzymatic activities of superoxide dismutase (SOD)-reduced glutathione (GSH) and catalase (CAT) were measured using commercial kits (Nanjing Jiancheng Bioengineering Institute, Nanjing, China). The protein concentrations were determined using the BCA assay kit (Vazyme Biotechnology, Nanjing, China). The level of MDA was estimated by evaluating the thiobarbituric acid reactive substances (TBARS) at 532 nm. SOD activity was detected based on its ability to inhibit the oxidation of oxymine by the xanthine–xanthine oxidase system. The GSH concentration was evaluated using a spectrophotometric kit (Biodiagnostic, Egypt). This method is based on the fact that the sulfhydryl component of GSH reacts with 5,5-dithiobis-2-nitrobenzoic acid (Ellman’s reagent) to produce 5-thio-2-nitrobenzoic acid, which has a yellow color. The 5-thio-2-nitrobenzoic acid was measured colorimetrically at 420 nm. CAT activity was determined by measuring the decrease in absorbance at 405 nm due to H_2_O_2_ dismutation.

### 2.6 Histopathologic evaluation by light microscopy

The testis specimens were embedded in paraffin blocks after fixing in Bouin’s solution. Sections of thickness 4 μm were obtained and stained using hematoxylin and eosin (H&E). Histopathological changes in the seminiferous tubules were observed using an ocular micrometer (Olympus, Tokyo, Japan) at a magnification of ×400, and images were obtained. The testicular injury and spermatogenesis were assessed histopathologically using Johnsen’s mean testicular biopsy score (MTBS) criteria (Johnsen, 1970).

### 2.7 Transmission electron microscopy analysis

TM4 cell pellets were fixed with 2.5% glutaraldehyde (Lab-coms) at room temperature for 2 h and then overnight at 4°C for 24 h. The fixed cells were then dehydrated with ethanol and embedded in ultrathin sections. Then, the sections were counterstained with uranyl acetate and lead citrate. The TM4 cell ultrastructure was investigated via transmission electron microscopy (TEM, Hitachi, Tokyo, Japan) at ×15,000.

### 2.8 Detection of apoptosis by flow cytometry

The cell apoptosis was measured by flow cytometry. TM4 cells after OGD/R induction and treatment with SAL were collected and double-stained with Annexin V-FITC and PI according to the assay kit (Wuhan BestBio^®^ Institute, Wuhan, China). Apoptosis of the cells was measured and analyzed using a ParTec flow cytometer (Germany) and ParTec Software.

### 2.9 Cell JC-1 detection

The TM4 cells were cultured, and OGD/R model was constructed. The corresponding groups were then treated with 0.05 mmol/L SAL for 24 h, following which cell samples were collected for staining and JC-1 was detected as per the instructions of the JC-1 detection kit. The green fluorescence of the JC-1 monomer (cytoplasm) was collected from the FITC channel, and the red fluorescence of the J-aggregates (mitochondria) was collected from the PE channel. The ratio of average fluorescence intensities of FITC to PE was used to judge the degree of injury.

### 2.10 RNA-seq assay and transcriptomic analysis

The total RNA was extracted from samples, and the concentration and purity of the extracted RNA were detected using the Nanodrop 2000; the RIN value was also determined using Agilent2100. The oligo dT enriched and randomly fragmented mRNA were detected along with isolated small fragments of approximately 300 bp using magnetic beads. In the presence of reverse transcriptase, six-base random primers were added, and the mRNA was used as a template to synthesize one-strand cDNA, followed by two-strand synthesis to form a stable double-stranded structure that was connected to the adaptor and sequenced using the second-generation high-throughput sequencing platform. The filtered sequencing of each sample was compared with the reference genome to identify its location. The transcripts were compared with the reference book using Cuffcompare to evaluate the construction of the transcript and discover new unknown genes. New genes with coding potential were found using CPC2, and BLAST software was used to compare the sequences of the newly discovered genes with the Swiss-Prot, GO, eggNOG/COG, Kog, KEGG, and PFAM databases to obtain their annotation information.

HTSeq was used to count the read values of each of the genes as the original expressions of the genes. To ensure that the number of fragments truly reflected the transcript expression level, the fragments per kilobase of transcript per million fragments mapped (FPKM) was used to standardize the expression level, and DESeq software was used to analyze the gene expression levels between two comparison groups. DESeq was also used to analyze the differences in gene expressions, and the conditions for screening differentially expressed genes were as follows: |log2FoldChange| > 1, with significance *p* < 0.05. GO enrichment analysis and KEGG pathway analysis were carried out on the differential genes, and the pathway for *p* < 0.05 in each GO classification and KEGG pathway was analyzed. The raw sequence data (RNA-seq) in this work have been deposited in the Genome Sequence Archive (Genomics, Proteomics & Bioinformatics 2021) at the National Genomics Data Center (Nucleic Acids Res 2022), China National Center for Bioinformation/Beijing Institute of Genomics, Chinese Academy of Sciences (GSA: CRA015453) and are publicly accessible at https://ngdc.cncb.ac.cn/gsa.

### 2.11 Metabolomics analysis

To extract the metabolites, approximately 250 μL of water was added to the sample before placing in a liquid nitrogen tank for 1 min; the sample was then retrieved, thawed, mixed evenly for 30 s, and ultrasonically treated in an ice water bath for 10 min, following which a 50 μL sample of the homogenate was removed for BCA protein quantification. Here, approximately 800 μL methanol was added and acetonitrile at 1:1(V/V) was added to the remaining 200 μL sample before transferring to a 2 mL EP tube, ultrasonicating in an ice water bath for 10 min, and allowing to stand at -40°C for 1 h; the sample was next centrifuged at 4°C and 12,000 rpm for 15 min, and the supernatant was removed as the QC sample. The tissue samples were divided into three groups and analyzed for metabonomics using LC-MS. For the polar metabolites, the Vanquish ultra-performance liquid chromatograph (Thermo Fisher Scientific) was used in this project, and the target compounds were separated using a Waters ACQUITY UPLC BEH Amide liquid chromatography column. An Orbitrap Exploris 120 mass spectrometer was used to collect the primary and secondary mass spectrometry data using the control software application Xcalibur (version: 4.4, Thermo).

SIMCA software (v16.0.2, Sartorius Stedim Data Analytics AB, Umea, Sweden) was used to perform the LOG conversion and CTR formatting on the data before automatic modeling and analysis. The VIP value was calculated using SIMCA, and metabolites with VIP > 1 were screened. Once the ionic strengths of the metabolites were standardized, the metabolites with *p* < 0.05 were screened by t-test and variance analysis. The intersection of metabolites with VIP > 1 and *p* < 0.05 was selected as the differential metabolite. Principal component analysis (PCA) and orthogonal partial least-squares discriminant analysis (OPLS-DA) were applied using SIMCA software. The KEGG IDs of the differential metabolites were introduced into MetaboAnalyst (https://www.metaboanalyst.ca/) for enrichment, and the metabolic pathway after administration was analyzed. The metabolomics raw data are deposited in the OMIX database under accession number OMIX006033.

### 2.12 Liposome transfection

The TM4 cells were cultured routinely, and the transfection began when the confluence of cells reached 70%. The transfection solution for the 96-well-plate cultured cells was prepared according to the instructions of the Lipofectamine^TM^3000 transfection kit, in which component I consisted of 0.1 µg of Nfr2 plasmid DNA, 0.2 µL of P3000TM reagent, and 5 µL of Opti-MEMTM medium. Component II consisted of 0.3 µL of LipofectamineTM3000 reagent and 5 µL of Opti-MEMTM medium. After preparing components I and II, they were allowed to stand at room temperature for 5 min before being mixed together and incubated at room temperature for 20 min. The mixed solution was gently added dropwise to the culture solution of cells to be transfected; the mixture was shaken lightly and mixed evenly, and the culture was continued for an additional 48 h.

### 2.13 Western blot

The testicular tissues and treated TM4 cells were grounded in a 1:10 (w/v) lysis buffer (Nanjing KeyGen Biotech Co., Ltd., Nanjing, China). The homogenates were processed in a refrigerated centrifuge at 12,000 rpm and 4°C for 15 min, and the supernatant was obtained and used to detect the total protein levels. The protein concentration was analyzed using a BCA protein assay kit (Thermo, United States of America). The samples (30 μL) at each protein concentration were subjected to electrophoresis with 12% and 8% SDS/PAGE and transferred to a polypropylene fluoride membrane (200 mA, 2 h). The membrane was blocked with 5% milk (2.5 g of skimmed milk powder: 50 mL of PBST) for 2 h at room temperature. After combining with a certain proportion of antibodies, the membrane was washed for 5 min, incubated with secondary antibodies for 2 h at room temperature, and washed with a secondary antibody. The protein band was visualized using an ECL kit, and the density of each band was quantified with a Western blot detection system (Quantity One software; Bio-Rad Laboratories, Hercules, CA, United States of America).

### 2.14 Reverse transcription qPCR (RT-qPCR)

The total RNA was extracted in accordance with the instructions for commercial reagents (Axygen, China). The expressions of GPX4 and TFR1 mRNA were detected using RT-qPCR. The RNA was reverse-transcribed with Trans Script First-Strand cDNA Synthesis SuperMix. The following are the primers used. GPX4 forward primer: CTC GCA ATG AGG CAA AAC TGA CG; GPX4 reverse primer: TCC TTG ATT TCT TGA TTA CTT CCT GGC T; TFR1 forward primer: GCT CGT GGA GAC TAC TTC CGT GC, TFR1 reverse primer: CTT GGA GAT ACA TAG GGC GAC AGG; GAPDH forward primer: CGG TGC TGA GTA TGT CGT GGA GTC; GAPDH reverse primer: GGC GGA GAT GAC CCT TTT G. The relative changes in the expression of the amplified gene were determined using a comparative cycle threshold (Ct) method with 2^−ΔΔCT^. The relative expressions of GPX4 and TFR1 gene were normalized with respect to that of GAPDH, which was regarded as the endogenous control. The fold induction (2^−ΔΔCT^) was represented by the calculated result.

### 2.15 Statistical analysis

Statistical analyses were performed using GraphPad Prism 8.0 and SPSS 24.0 statistical software. The data were statistically evaluated using the one-way analysis of variance (ANOVA), and all values were expressed as the mean ± standard deviation. The significance between the groups was determined using the Student’s paired t-test, and the values were considered significant at *p* < 0.05.

## 3 Results

### 3.1 Protective effect of SAL on testicular I/R injury

First, the pathological condition of the testicular tissue was studied, and the testicular histopathological changes (H&E) and Johnsen’s scores are presented in Figure 1. The testes of the control mice show the presence of regular seminiferous tubular morphologies with normal spermatogenesis and normal testicular architectures (Figure 1C). The testicular tissues of the I/R mice show seminiferous tubule atrophy and severe damage, disordered arrangement of sperm cells differentiation, and stagnation of development at all levels; the sperm cells show interstitial tissue damage and loss, supporting the decrease in the number of cells, markedly increased cavity structure, and significant decrease in the tube cavity sperm cells (Figure 1C). Moreover, in the I/R mice, numerous vacuoles were spread around the testicular cells with various degrees of spermatogenetic arrest, and the Johnsen’s scores were significantly lower than those of the control group (Figure 1D). In H&E staining, the SAL (100 mg/kg) treatment group showed the best results for testicular tissue recovery in I/R mice. The spermatogenic cells were arranged tightly and neatly, with visible cells at different stages or in the process of differentiation. The Johnsen’s scores were significantly higher (Figure 1D). In addition, TUNEL staining was used to observe the apoptosis of the testicular spermatogenic cells. Compared with the normal group, the degree of apoptosis of the spermatogenic cells in I/R group increased significantly, and the degree of apoptosis decreased significantly after SAL (100 mg/kg) treatment (Figure 1E).

To mimic the testicular I/R injuries, the TM4 cells were exposed to OGD/R as an *in vitro* model. The cell viability was determined using CCK-8. Compared with the control group, the viability of the TM4 cells decreased significantly after OGD/R treatment. In contrast, SAL treatment increased the cell viability (0.05 mmol/L) (Figure 1F). Moreover, the apoptosis of TM4 cells was detected using the mitochondrial membrane potential and flow cytometry; the results show that compared with the control group, the apoptosis rate of TM4 cells increase significantly after OGD/R treatment (Figure 1G, H). On the contrary, SAL treatment reduces the apoptosis rate of the TM4 cells (Figure 1G, H). These data imply that SAL has a certain protective effect on testicular I/R injury.

### 3.2 SAL alleviates oxidative stress *in vivo* and *in vitro*

**FIGURE 2:**
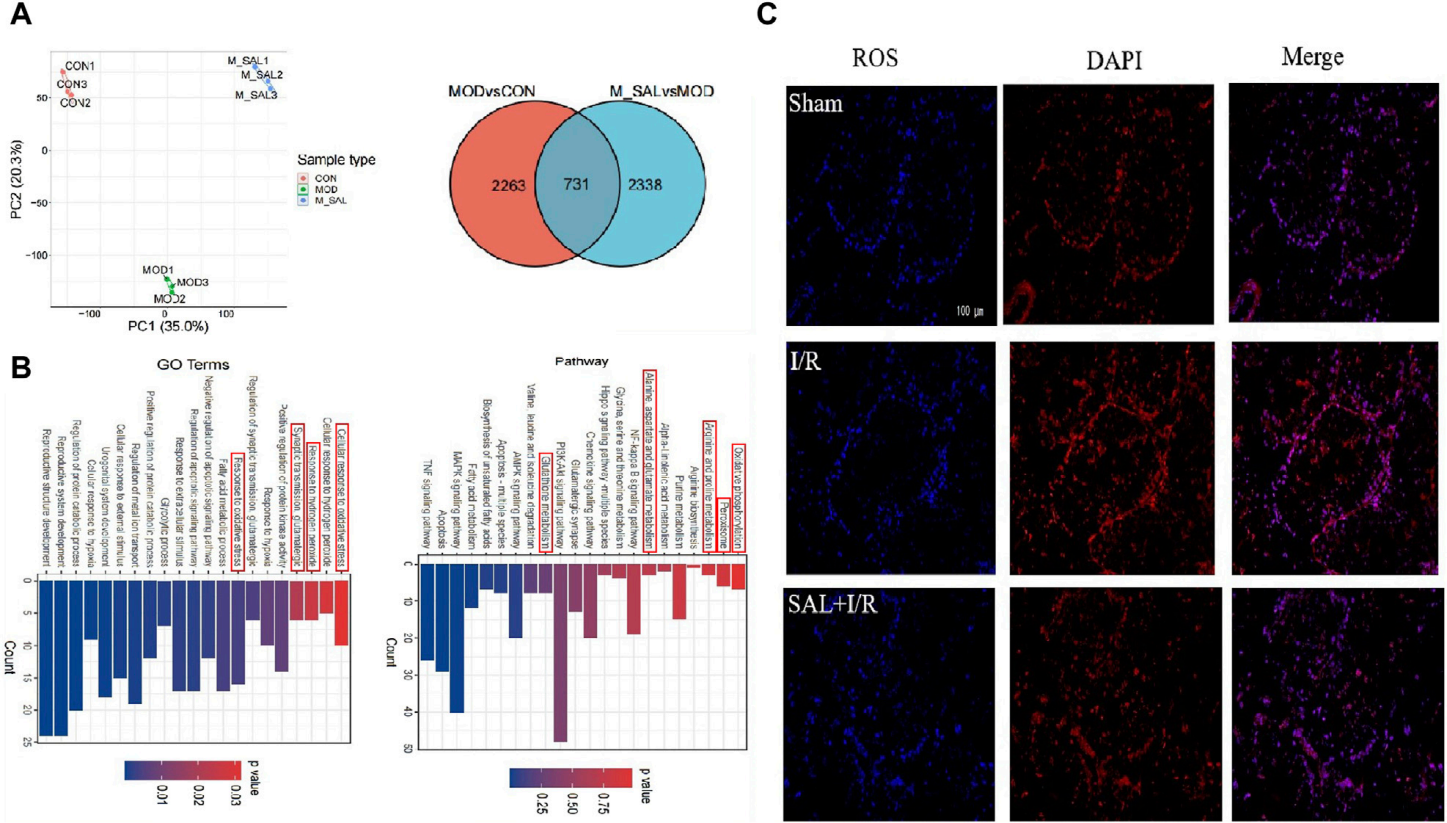
Transcriptomics analysis of SAL: **(A)** between-group PCA diagram and Venn diagram of DEGs; **(B)** KEGG enrichment analysis for the DGEs of I/R + SAL vs. I/R; **(C)** fluorescence staining of ROS.

To study the possible mechanism of SAL’s protective effect on testicular I/R injury, transcriptomics analysis was applied. First, the samples were analyzed using PCA and cluster analysis to measure the expression levels of each of the samples from the perspective of the overall dispersion of expression, with the abscissa representing the different samples and ordinate representing the logarithmic values of sample expression FPKM. The PCA score chart showed that there were obvious separations between the control and I/R groups as well as the I/R and SAL groups (Figure 2A).

DESeq was used to analyze the differences in gene expressions, and the conditions for screening differentially expressed genes were |log2FoldChange| > 1 and significant *p* < 0.05. Compared with the control group, approximately 2,263 transcripts in the I/R group were obviously out of balance. Compared with the I/R group, there are 2,338 transcripts in the SAL group that were obviously out of balance. A total of 731 common DEGs were altered (Figure 2A). GO enrichment and KEGG pathway analysis were carried out on the highly significant DEGs to determine the enrichment degrees of the gene products in various GO categories and to determine the functions of the DEGs. The functions of these DEGs are based on the GO database and gene pathway with respect to the KEGG database. Through GO function analysis of the above genes, the related pathways in GO BP were found to include response to oxidative stress, fatty acid metabolic process, synaptic transmission, glutamatergic, regulation of apoptotic signaling pathway, cellular response to external stimulus, and cellular response to hypoxia. According to the KEGG database, the differentiated paths included oxidative phosphorylation; peroxisomes; arginine and proline metabolisms; alanine, aspartate, and glutamate metabolisms; NF-κ B signaling pathway; glutathione metabolism; biosynthesis of unsaturated fatty acids; and PI3K-Akt signaling pathway (Figure 2B). The enrichment changes in these pathways are closely related to SAL; among them, it has been reported that oxidative stress plays an important role in testicular I/R injury.

**FIGURE 3:**
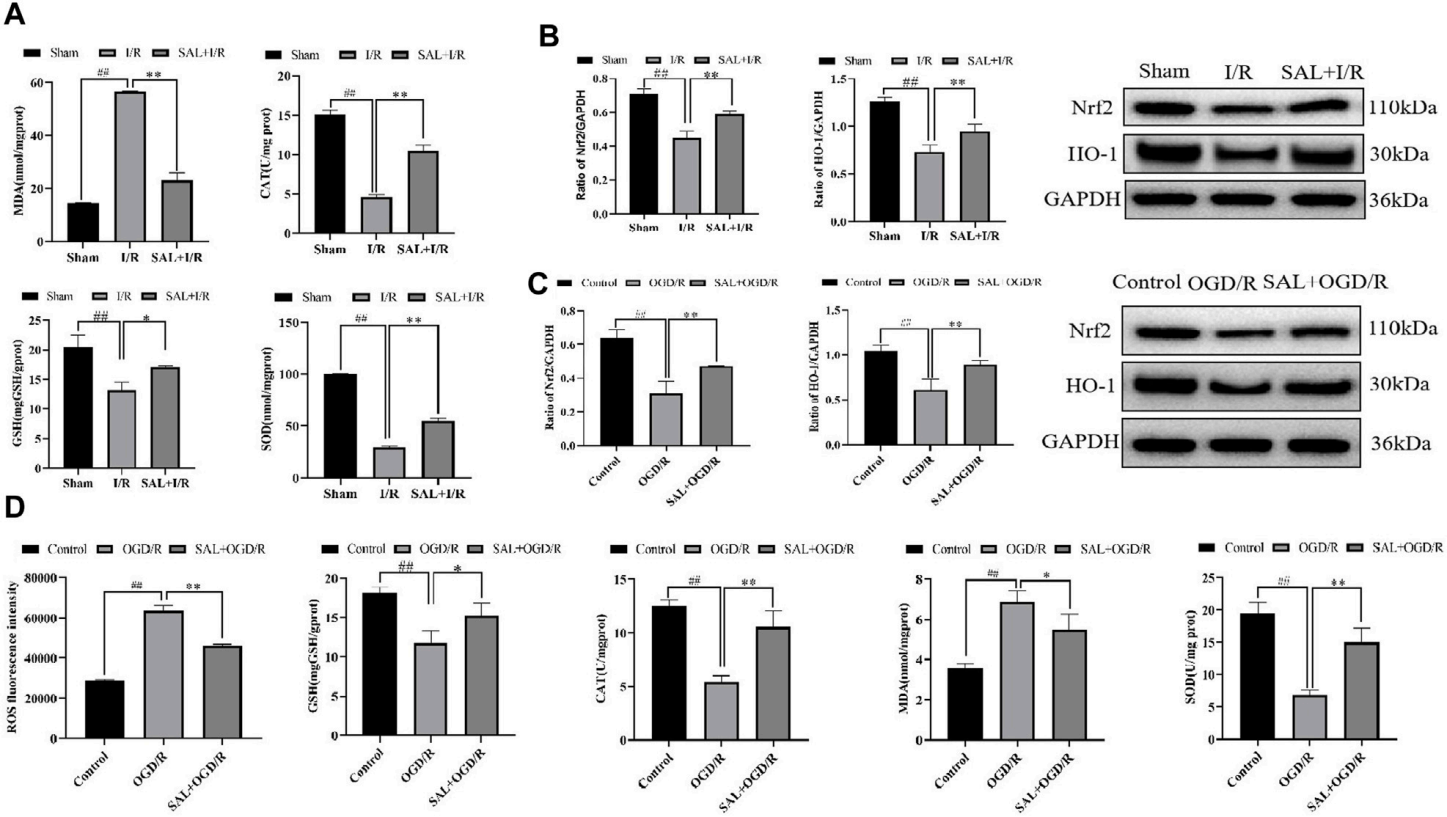
SAL alleviates oxidative stress *in vivo* and *in vitro*: **(A)** effects of SAL on the levels of MDA, SOD, CAT, and GSH in testicular I/R injury; **(B)** effects of SAL on the expressions of Nrf2 and HO-1 proteins in testicular I/R injury; **(C)** effects of SAL on the expressions of Nrf2 and HO-1 proteins in the TM4 Sertoli cell OGD/R injury; **(D)** effects of SAL on the levels of ROS, MDA, SOD, CAT, and GSH in the TM4 Sertoli cell OGD/R injury. Each value indicates the mean ± standard deviation (*n = 6*)*.* ^*##*^ *p < 0.01* vs. sham group or control group; *p < 0.05; **p < 0.01 vs. I/R group or OGD/R group.

To further study the effects of SAL on oxidative stress in testicular I/R injury, the animal I/R and OGD/R cell models were used to detect the expressions of indexes related to oxidative stress. In the animal I/R model, the ROS expression was observed by immunofluorescence, where the fluorescence intensity was weak in the control group but intense expression of ROS was induced in the I/R group. However, oral administration of SAL (10 mg/kg) significantly decreased the ROS immunoreactivity in the testes (Figure 2C). According to the biochemical results, compared with the control group, the levels of MDA in the I/R group increased obviously while the levels of CAT, SOD, and GSH decreased obviously (Figure 3A). The MDA level decreased significantly and the levels of CAT, SOD, and GSH increased significantly after SAL (100 mg/kg) treatment (Figure 3A). The results from the Western blot analysis demonstrated that the Nrf2 and HO-1 protein levels in the I/R group were significantly reduced compared with those of the control group (Figure 3B). The administration of SAL (100 mg/kg) to I/R mice produced marked increases in Nrf2 and HO-1 protein expressions (Figure 3B).

To mimic testicular I/R injury, TM4 cells were exposed to OGD/R as an *in vitro* model. Intracellular ROS generation was determined according to DCFH-HA, and exposure to OGD/R caused excessive ROS production while treatment with SAL (0.05 mmol/L) reduced the ROS levels (Figure 3D). The biochemical results showed that compared with the control group, the level of MDA in the OGD/R group increased significantly while the levels of CAT, SOD, and GSH decreased significantly (Figure 3D). After treatment with SAL (100 mg/kg), the MDA level decreased significantly while the CAT, SOD, and GSH levels increased significantly (Figure 3D). According to the protein blot analysis, compared with the control group, the Nrf2 and HO-1 protein levels in the OGD/R group were significantly lower (Figure 3C). The administration of SAL (0.05 mmol/L) to OGD/R mice resulted in significant increases in the expressions of Nrf2 and HO-1 proteins (Figure 3C). These data indicate that the protective effects of SAL on testicular I/R injury are related to oxidative stress.

### 3.3 SAL inhibits occurrence of ferroptosis *in vivo* and *in vitro*

**FIGURE 4:**
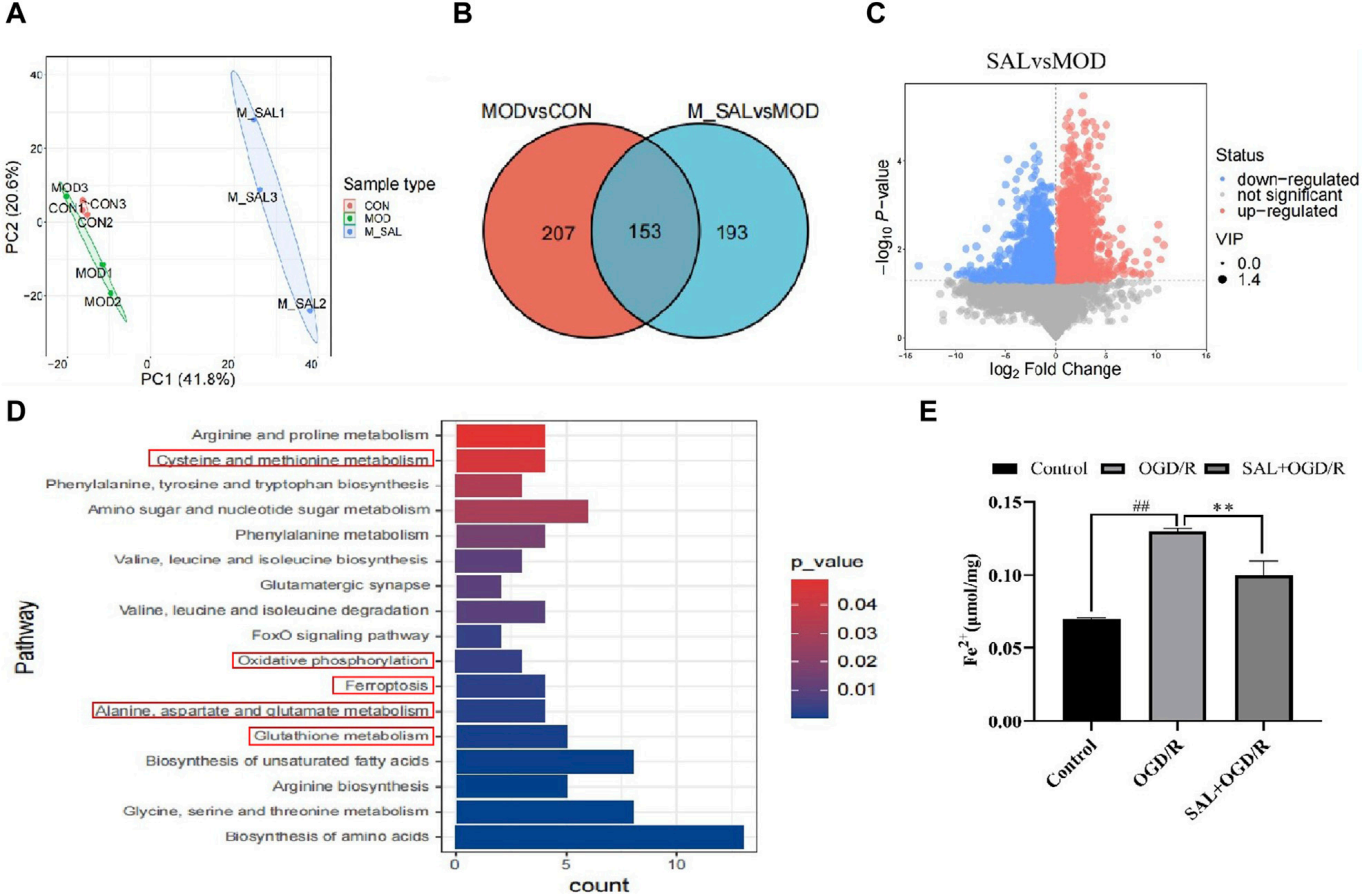
Metabonomics analysis of SAL: **(A)** between-group PCA diagram; **(B)** Venn diagram of the SCMs; **(C)** volcano map of I/R + SAL vs. I/R; **(D)** KEGG enrichment analysis of I/R + SAL vs. I/R; **(E)** effect of SAL on the level of Fe^2+^ in the TM4 Sertoli cell OGD/R injury. Each value represents the mean ± standard deviation (n = 6). ^##^ *p* < 0.01 vs. control group; **p < 0.01 vs. OGD/R group.

It has been reported that oxidative stress is closely related to ferroptosis. To further study the relationship between oxidative stress and ferroptosis, metabonomics was used for analysis. The score scatter plot for the PCA model with QC shows each scatter representing a sample, where the color and shape of the scatter represent different groups. When the distribution of the sample points is closer, the species and contents of the metabolites in the sample are more similar. Conversely, when the sample points are farther, the difference in the overall metabolic level is greater. All samples are within 95% confidence interval (Hotelling’s T-squared ellipse) (Figure 4A).

The conditions for screening the differentially expressed genes are VIP >1 and *p*-value < 0.05. Compared to the normal group, 207 metabolites in the I/R group were obviously out of balance. Compared to the I/R group, 193 metabolites in the SAL group were obviously out of balance and 153 metabolites changed together (Figure 4B). Through path enrichment analysis, it was found that the path with *p* < 0.05 was the most relevant path. The main metabolic pathways co-enriched in the SAL group include glutathione metabolism; alanine, aspartate, and glutamate metabolisms; oxidative photolysis; ferroptosis; glycine, serine, and threonine metabolisms; and cystine and methionine metabolisms (Figure 4C). Transcriptomics and metabonomics analyses thus show that SAL regulation is closely related to oxidative stress and ferroptosis.

**FIGURE 5:**
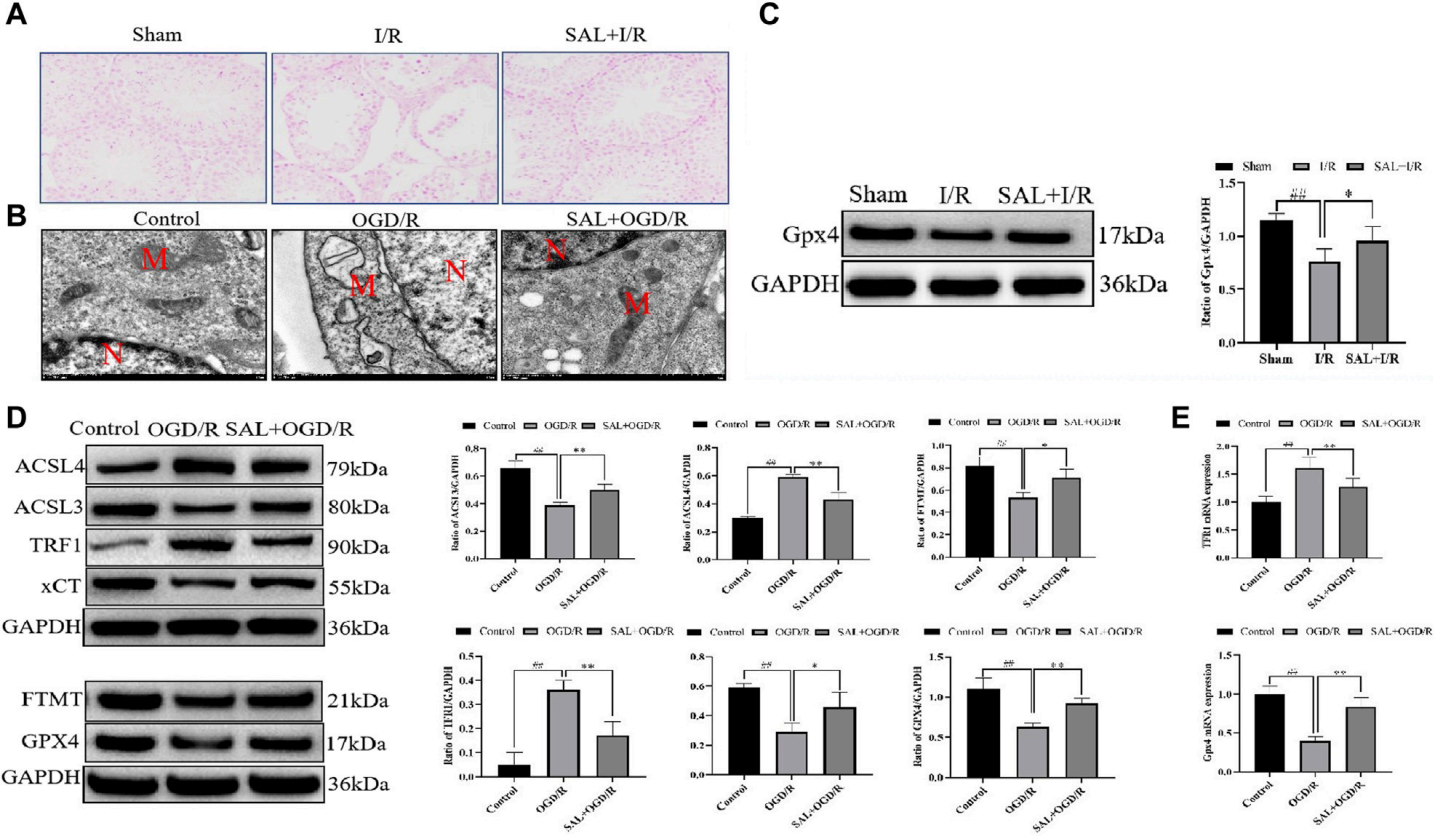
SAL inhibits ferroptosis *in vivo* and *in vitro*: **(A)** effect of SAL on the iron stain in testicular I/R injury; **(B)** ultrastructure of the TM4 cells in each group, as analyzed by TEM, where N is the nucleus and M is the mitochondrion; **(C)** effect of SAL on the expression of GPX4 in testicular I/R injury; **(D)** effects of SAL on the expressions of ACSL4, TRF1, ACSL3, FTMT, xCT, and GPX4 in the TM4 Sertoli cell OGD/R injury; **(E)** effects of SAL on the expressions of TRF1 and GPX4 mRNA in the TM4 Sertoli cell OGD/R injury. Each value indicates the mean ± standard deviation (*n = 6*)*.* ^##^ *p < 0.01* vs. sham group or control group; *p < 0.05; **p < 0.01 vs. I/R group or OGD/R group.

The relationship between oxidative stress and ferroptosis in I/R injuries was further studied. The expressions of ferroptosis-related indexes were detected using the animal I/R and OGD/R cell models. Using immunofluorescence, the expression of Fe^2+^ was observed. In the I/R group, the number of positive cells increased significantly, and after treatment with SAL at a dose of 100 mg/kg, the number of positive cells decreased significantly (Figure 4E). The results from the Western blot analysis demonstrated that the GPX4 protein level in the I/R group was significantly lower than that in the control group (Figure 5C). The administration of SAL (100 mg/kg) to I/R mice produced a marked increase in GPX4 protein expression (Figure 5C).

To mimic testicular I/R injury, the TM4 cells were exposed to OGD/R as an *in vitro* model. TEM analysis of the OGD/R group showed the typical morphology of the ferroptosis cells, which are characterized by smaller than normal mitochondria, increased mitochondrial membrane density, and mitochondrial cristae disappearance or dissolution. However, these features reduced upon SAL treatment (0.05 mmol/L) (Figure 5B). The results from the Western blot analysis demonstrated that the ACSL3, FTMT, xCT, and GPX4 protein levels in the OGD/R group were significantly reduced compared with those of the control group (Figure 5D). The administration of SAL (0.05 mmol/L) to the OGD/R group produced a marked increase in the ACSL3, FTMT, xCT, and GPX4 protein expressions (Figure 5D). Similarly, the ACSL4 and TRF1 protein levels in the OGD/R group markedly increased compared with those of the control group (Figure 5D). The administration of SAL (0.05 mmol/L) to the OGD/R group significantly reduced ACSL4 and TRF1 protein expressions (Figure 5D). The qRT-PCR study showed that the relative expression of the TRF1 mRNA increased significantly in OGD/R group, while the relative expression of the GPX4 mRNA decreased significantly. SAL (0.05 mmol/L) significantly decreased the relative expression of TRF1 mRNA and increased that of GPX4 mRNA (Figure 5E). All these data suggest that SAL may protect against testicular I/R injuries by regulating oxidative stress and ferroptosis.

### 3.4 Verification of the protective mechanism of SAL on testicular injury

**FIGURE 6:**
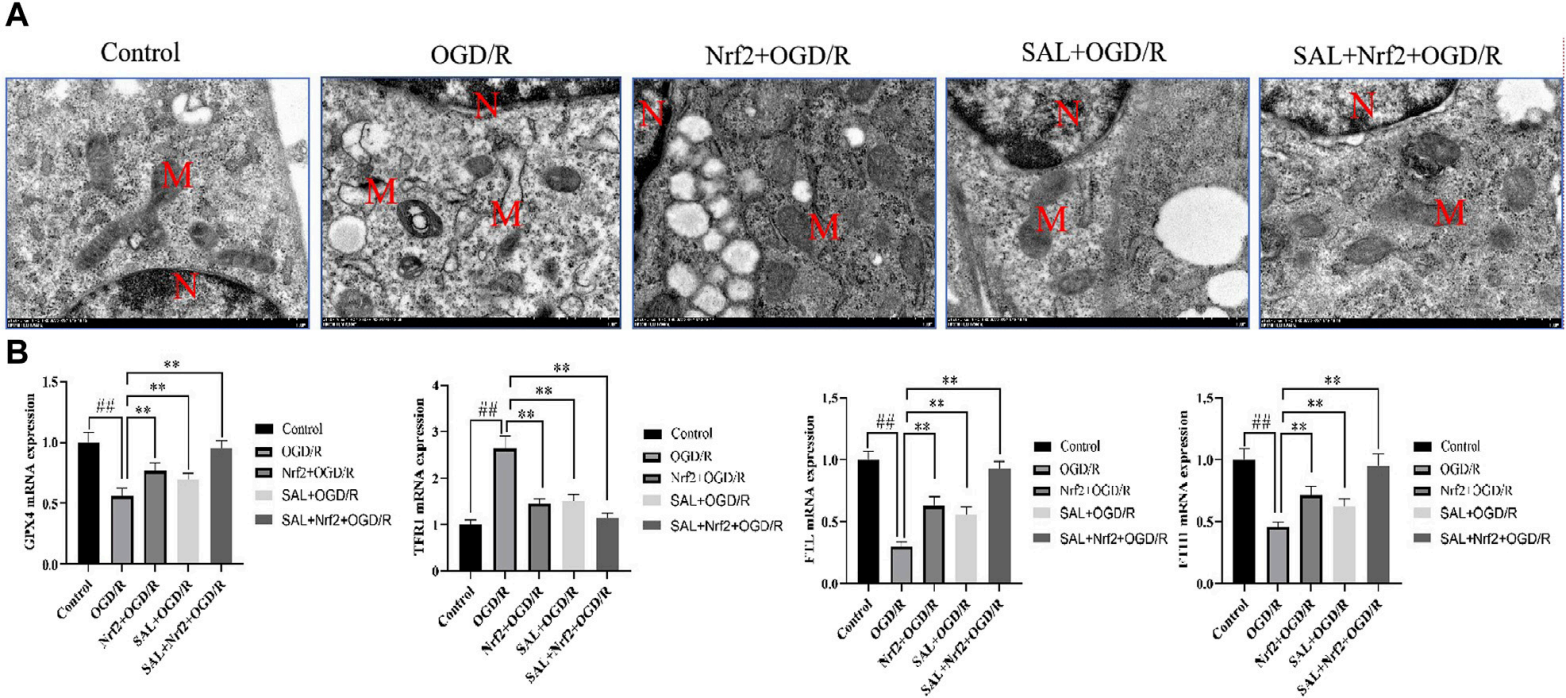
Effects of Nfr2 overexpression in the OGD/R injury of TM4 Sertoli cells: **(A)** ultrastructure of the TM4 cells in each group, as analyzed by TEM, where N is the nucleus and M is the mitochondrion; **(B)** effects of SAL on the expressions of HO-1, GPX4, FTH1, and TRF1 mRNA in the TM4 Sertoli cells in OGD/R injury. Each value indicates the mean ± standard deviation (*n = 6*)*.* ^*##*^ *p < 0.01* vs. control group; *p < 0.05; **p < 0.01 vs. OGD/R group.

To verify the target of SAL in the treatment of I/R injury, the Nfr2 plasmid was constructed and transfected into TM4 cells by liposomes, and the changes in the related indexes after Nrf2 overexpression were observed. TEM analysis showed the typical morphology of the ferroptosis cells in the OGD/R group, which was characterized by smaller than normal mitochondria, increased mitochondrial membrane density, and mitochondrial cristae disappearance or dissolution. However, the typical symptoms of ferroptosis were obviously improved in the Nrf2 transfection and SAL treatment groups and especially in the Nrf2 transfection + SAL treatment group (Figure 6A). In the Nrf2 transfection and SAL treatment groups, the mRNA expressions of HO-1, GPX4, and FTH1 decrease significantly while the mRNA expression of TRF1 increases significantly in OGD/R injury of the testicular TM4 cells. However, the mRNA expressions of these related proteins change more obviously in the Nrf2 transfection + SAL treatment group (Figure 6B). These results show that SAL can protect against testicular I/R injury by regulating the Nfr2/HO-1/GPX4 signaling pathway to inhibit ferroptosis.

## 4 Discussion

Testicular dysfunction associated with male infertility is one of the serious side effects of testicular torsion (Hansen et al., 2023; Minas et al., 2023; Mohamed et al., 2023). Testicular torsion leads to impaired testicular perfusion by arterial obstruction and subsequently ischemic necrosis (Guazzone and Lustig, 2023; Yi et al., 2023). The management of this urological emergency requires immediate detorsion of the twisted testis. Detorsion results in reperfusion of the ischemic tissue, and this process is actually an I/R injury that triggers further biochemical and morphological alterations, such as excessive ROS production and LPO, along with excessive triggering of a cytokine storm as well as DNA and cellular apoptotic damage (Ozbek et al., 2015; Liu et al., 2022; Törzsök et al., 2022). However, treatments to protect against testicular I/R injury are gradually attracting increased attention while still remaining unsolved. In the present study, the protective effect and potential mechanism of SAL on testicular I/R injury was confirmed for the first time *in vivo* and *in vitro*.

In the I/R damage model of the pharmacodynamic experiment, the testicular tissue morphology showed obvious damage, including reduced number of seminiferous tubules, disordered and different degrees of spermatogenic cells, vacuolization in the spermatogenic cells, and inhibited spermatogenesis. Consequently, the sperm parameters were seriously affected. Similar results were also observed in this research. However, after SAL treatment, the spermatogenic cells were arranged tightly and neatly with visually observable cells at different stages or in the process of differentiation. The apoptosis of spermatogenic cells was also improved significantly. In the *in vitro* model of OGD/R, it was observed that the apoptosis rate of the spermatogenic cells increased obviously. Previous studies have shown that inhibiting the apoptosis of spermatogenic cells can alleviate the I/R damage of testicular tissue. In this research, SAL significantly reduced the apoptosis rate of the spermatogenic cells. Therefore, these results provided new evidence for the protective effects of SAL on spermatogenic cells. Nevertheless, the *in vivo* molecular mechanism by which SAL exerted protective effects remains a key point.

The transcriptome represents an inevitable link between the genetic information of the genome and the biological function of the proteome (Zhi et al., 2024). Regulation at the transcriptional level is the most pivotal pattern of self-regulation (Ou et al., 2024). Transcriptomic analysis of the testicular tissue through next-generation sequencing (RNA-seq) allows high-throughput and detailed characterizations of the gene-expression profiles at the tissue level (Hu et al., 2023). Among the complex and diverse mechanisms of action, this technology may enable finding the most relevant mechanism for protection. Transcriptomic results of the testicular tissue suggest that 731 differentially expressed genes could be identified among the I/R + SAL vs. I/R groups, indicating the effects of drug therapy rather than single or multiple genes. In GO BP, the related pathways include response to oxidative stress, fatty acid metabolic process, glutamatergic, regulation of the apoptotic signaling pathway, and cellular response to hypoxia. Through analysis, it was found that oxidative stress played a vital role in the treatment of diseases. To further elucidate the potential mechanism of SAL therapy, differentially expressed genes in the testis were enriched by KEGG. As mentioned above, the common signal pathways of the transcriptome in the KEGG-pathway-enrichment analysis include the following: oxidative phosphorylation; peroxisomes; alanine, aspartate, and glutamate metabolism; and other signal pathways. These predictors show that SAL has outstanding effectiveness in resisting diseases and oxidative stress damage. According to a comprehensive analysis of the above results, it was also found that oxidative stress may be an important potential factor in inducing I/R damage. High levels of oxygen free radicals are produced during I/R of testicular tissue, seriously damaging the sperm membrane and affecting sperm quality (Zhong et al., 2020; Ning et al., 2021). Nrf2 is a key transcription factor for regulating ROS and is also an important regulator for maintaining intracellular redox balance (Chen et al., 2024; Wang et al., 2024). Nrf2 can also induce and regulate the composition and expression of antioxidant proteins (including CAT, SOD, and GSH), reduce the production of ROS, and maintain the redox stability of the body (Sang et al., 2024; Zhang et al., 2024). Studies have shown that testicular I/R damage is related to the decrease in antioxidant enzymes regulated by Nrf2 (Antonuccio et al., 2022). Indeed, the use of some antioxidants (including SAL) has been extensively discussed for preventing testicular injury (Matzkin et al., 2021; Cai et al., 2023). To verify this, the present work shows that SAL therapy can protect the activities of CAT, SOD, and GSH in the testes and increase the expressions of Nrf2 and HO-1 in the animal I/R and OGD/R cell models. SAL also reduces the levels of ROS and MDA. Therefore, SAL is an effective antioxidant that can protect the integrity of the spermatogenic cell membrane and inhibit oxidative stress.

The key iron-storing proteins ferritin light chain/ferritin heavy chain (FTL/FTH1) and GPX4 are regulated by Nrf2. Regulating LPO and ferroptosis by targeting Nrf2 is hence a feasible disease-intervention strategy (Long et al., 2023; Qiu et al., 2023). Studies have shown that oxidative stress is closely related to ferroptosis (Kirbas et al., 2024). The main characteristics of ferroptosis are intracellular iron overload, significantly increased ROS and LPO levels, and a final lipid peroxidation reaction inside the cell-membrane phospholipids that leads to membrane damage and cell death. Ferroptosis is defined as regulatory cell death caused by oxidative imbalance in the intracellular microenvironment. When the cystine transporter is inhibited, it gradually consumes intracellular GSH. Eventually, the core regulatory factor GPX4 is inactivated and LPO accumulates, which can induce cell death to a certain extent (Chen et al., 2024; Gao et al., 2024; Huang et al., 2024).

The metabonomic results suggest that 153 differentially expressed genes could be identified among the I/R + SAL vs. I/R groups. Enrichment analysis reveals that the main metabolic pathways of the SAL group enrichment are as follows: glutathione metabolism; alanine, aspartate, and glutamate metabolisms; oxidative photolysis; ferroptosis; glycine, serine, and threonine metabolisms; cystine and methionine metabolisms. Cystine/glutamic acid can promote the synthesis of GSH, which is an essential auxiliary factor of GPX4; when this synthesis is blocked, GPX4 activity is reduced, the antioxidant capacity of the cells is reduced, and ferroptosis is promoted (Gao et al., 2024; Huang et al., 2024). GPX4 is a type of peroxidase that widely exists in organisms; it can protect cells from membrane LPO and has been proven to be a negative regulator of ferroptosis. Glutathione and oxidative photolysis are also closely related to oxidative stress and ferroptosis (Chen et al., 2024).

Metabonomics and transcriptomics show the complementarity of the metabolic pathways and gene changes. The analysis results suggest that SAL regulation is closely related to oxidative stress and ferroptosis. The related verifications were performed in this study, and SAL is found to reduce the iron-ion level and expression of GPX4 in testicular I/R injuries. Similarly, SAL improves the typical symptoms of ferroptosis in the TM4 cells, decreases the expression levels of ACSL4 and TRF1 in the OGD/R model, and significantly increases the expressions of ACSL3, FTMT, xCT, and GPX4. SAL can also improve the expression levels of GPX4 and TRF1 mRNA. The predicted targets were verified by metabonomics and transcriptomics, and the results show that SAL could be used to treat testicular I/R injuries by regulating the Nfr2/HO-1/GPX4 signaling pathway to inhibit ferroptosis.

**FIGURE 7:**
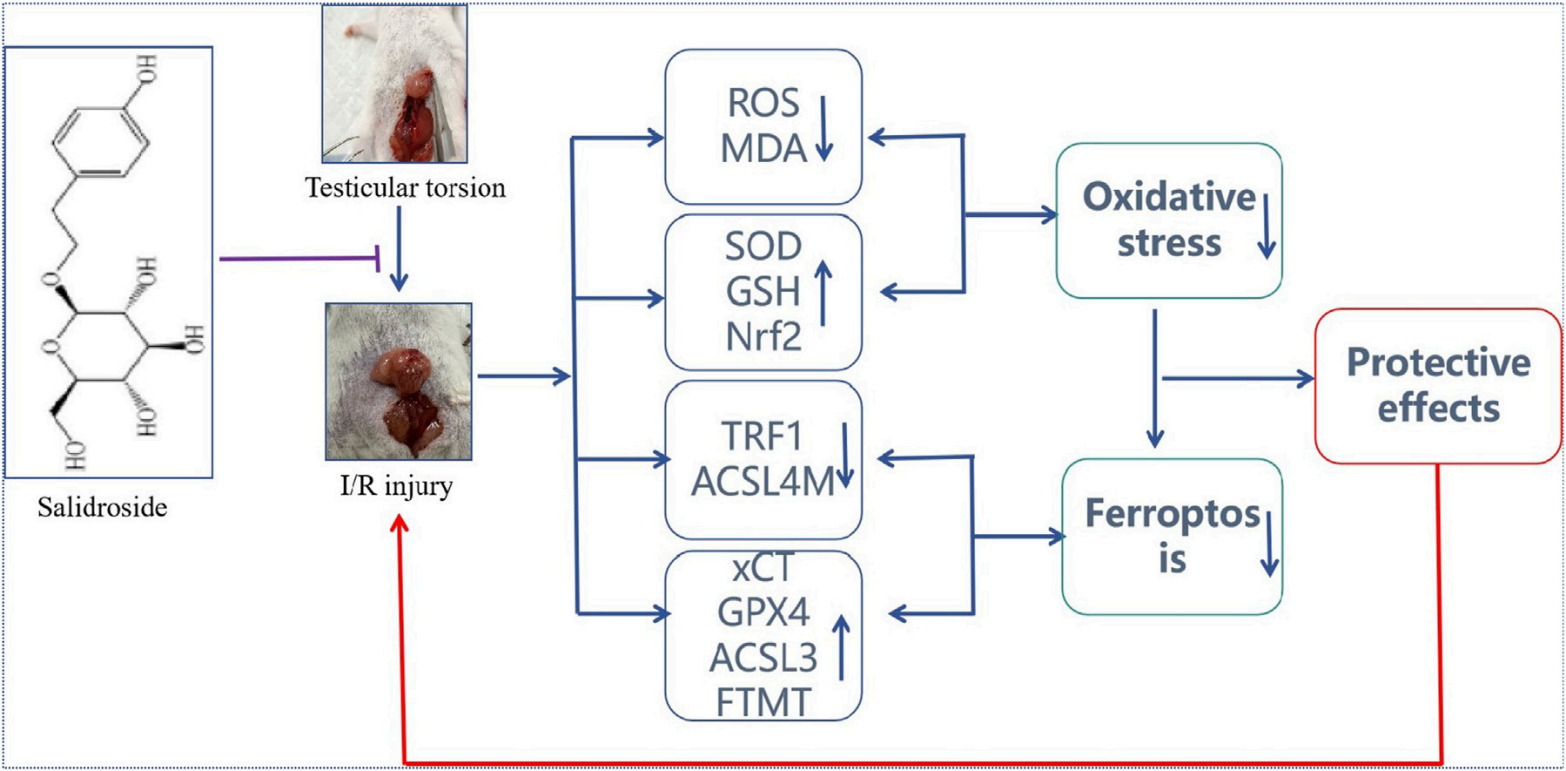
Protective effect of SAL on testicular I/R injury and its potential mechanism. SAL can protect against testicular I/R injury by regulating the Nfr2/HO-1/GPX4 signaling pathway to inhibit ferroptosis.

To further verify the target of SAL in treating I/R injuries, an Nfr2 plasmid that promotes overexpression of the Nrf2 gene in TM4 cells by liposome transfection was constructed in this study, and the effect of SAL on the Nfr2/HO-1/GPX4/ferroptosis pathway was observed. Overexpression of the Nrf2 gene can improve cerebral I/R injuries, especially in the treatment of acute myocardial infarction (Duan et al., 2019; Wang J. C. et al., 2020; Wang J. H. et al., 2020). The results of this study suggest that the mRNA expressions of HO-1, GPX4, and FTH1 decrease significantly and that TRF1 mRNA increase significantly in the OGD/R injury of testicular TM4 cells. In the Nrf2 transfection and SAL treatment groups, the mRNA expression levels of HO-1, GPX4, and FTH1 increased significantly, whereas TRF1 mRNA expression decreased significantly. The mRNA expressions of these related proteins change more obviously in the Nrf2 transfection + SAL treatment group. Moreover, the typical symptoms of ferroptosis are obviously improved in the Nrf2 transfection and SAL treatment groups, and the improvement in the Nrf2 transfection + SAL treatment group was more obvious. Therefore, SAL can protect against testicular I/R injury by regulating the Nfr2/HO-1/GPX4 signaling pathway to inhibit ferroptosis (Figure 7). However, the main drawback of this study is the flawed exploration of the mechanisms based on pharmacodynamics. First, there were no accurate analyses of the signaling pathways of SAL action by combined proteomic sequencing. Second, the key signaling pathways were not blocked, and the limited biotechnological tools used prevented sufficient exploration of the mechanisms. In the future, the explored methods may be combined with proteomics to determine the mechanism of SAL, which is crucial for effectively developing SAL for clinical applications.

In conclusion, this study clarifies the protective effects of SAL on testicular I/R injuries. Based on a comprehensive predictive analysis including transcriptomics and metabonomics, oxidative stress damage is the key mechanism in SAL treatment. Oxidative stress injuries can further induce ferroptosis of the testicular cells. Nevertheless, these predictions were verified in the OGD/R damage model of testicular cells. The pharmacological mechanism of SAL’s protective effects on testicular injuries was further verified by Nrf2 plasmid–liposome transfection. The results of this research showed that SAL intervention could prevent iron death by regulating the Nfr2/HO-1/GPX4 axis. All these results indicate that SAL has the potential for treating testicular I/R injuries.

## Data Availability

The raw sequence data RNA-seq in this paper have been deposited (GSA: CRA015453) and are publicly accessible at https://ngdc.cncb.ac.cn/gsa.
